# A CRISPR Resource for Individual, Combinatorial, or Multiplexed Gene Knockout

**DOI:** 10.1016/j.molcel.2017.06.030

**Published:** 2017-07-20

**Authors:** Nicolas Erard, Simon R.V. Knott, Gregory J. Hannon

**Affiliations:** 1 Cancer Research UK Cambridge Institute, University of Cambridge, Li Ka Shing Centre, Robinson Way, Cambridge CB2 0RE, UK; 2 Cold Spring Harbor Laboratory, 1 Bungtown Road, Cold Spring Harbor, NY 11724, USA; 3 Cedars-Sinai Medical Institute, 8700 Beverly Boulevard, Los Angeles, CA 90048, USA; 4 New York Genome Center, 101 6th Avenue, New York, NY 10013, USA

**Keywords:** CRISPR, sgRNA, algorithm, combinatorial, screen, expression, Cas9, prediction, lentivirus, library

## Abstract

We have combined a machine-learning approach with other strategies to optimize knockout efficiency with the CRISPR/Cas9 system. In addition, we have developed a multiplexed sgRNA expression strategy that promotes the functional ablation of single genes and allows for combinatorial targeting. These strategies have been combined to design and construct a genome-wide, sequence-verified, arrayed CRISPR library. This resource allows single-target or combinatorial genetic screens to be carried out at scale in a multiplexed or arrayed format. By conducting parallel loss-of-function screens, we compare our approach to existing sgRNA design and expression strategies.

## Highlights

• Target conservation and target-flanking homologous sequences impact sgRNA potency
• Functional impact is increased when Cas9 is focused to multiple sites in the target
• A resource for individual and combinatorial CRISPR screens is presented

## Graphical Abstract

**Figure:**
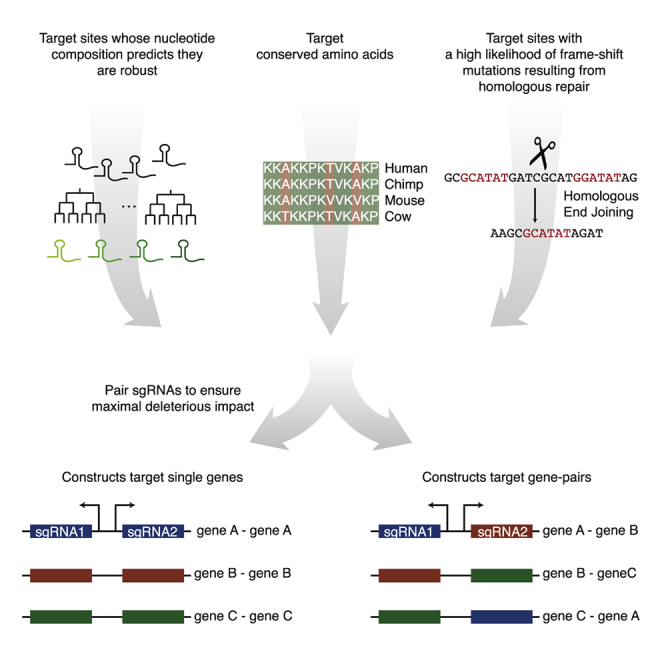

## Teaser

Erard et al. present an algorithm for predicting sgRNA potency that they combine with expression strategies to generate a CRISPR resource for performing individual, combinatorial, or multiplexed gene knockout in human cells. The resource is compared to other published tools through comparative multiplexed screens.

## Introduction

Genetic screens have played a fundamental role in charting genotype-phenotype interaction maps for a variety of organisms (Carpenter and Sabatini, 2004). However, confounding factors, such as non-uniformity in the efficacies of targeting molecules, have limited the depth to which data from such studies can be interpreted. These problems have been somewhat mitigated for short hairpin RNA (shRNA)-based gene silencing because, after several rounds of optimization, experimentally validated algorithms for selecting potent guide sequences have been developed (Fellmann et al., 2011, Knott et al., 2014, Pelossof et al., 2017). Similar approaches have been applied for selecting Cas9 guide RNAs (sgRNAs) for use with the type II clustered regularly interspaced short palindromic repeats (CRISPR) system, where large sgRNA potency datasets were used to train prediction algorithms (Chari et al., 2015, Doench et al., 2014, Doench et al., 2016). However, unlike with mRNA cleavage, Cas9-induced double-strand breaks (DSBs) leave a genomic scar whose characteristics determine the phenotypic consequences of targeting a locus.

The distance of the target from the translation start site is anti-correlated with sgRNA efficacy, probably because N terminus proximal frameshift mutations (FSMs) are more likely to induce nonsense-mediated mRNA decay or the production of truncated nonfunctional proteins (Doench et al., 2014). Non-homologous end joining (NHEJ) was thought to act as the predominant repair mechanism at Cas9-induced DSBs; this made predicting the likelihood of an FSM, for a given target, impossible. However, deep sequencing of these genomic scars has revealed that some homologous end joining (HEJ) contributes to repair of Cas9 cleavage events (Bae et al., 2014). Here the frequencies of specific repair resolutions are dependent on the length, guanine-cytosine (GC) content, and distance from the cut site of the two DSB-flanking homologous loci, suggesting that these likelihoods can be estimated. Finally, sgRNAs that focus Cas9 to functional domains provide a greater probability of phenotypic impact, likely because in-frame mutations in these regions have a greater potential to disrupt protein function (Shi et al., 2015).

The implementation of optimized effector expression strategies should also drive the efficacy of CRISPR knockout assays. Systems have been developed in which multiple RNA polymerase III promoters drive independent sgRNAs (Vidigal and Ventura, 2015). Alternatively, others have shown that Cpf1 can be focused to multiple targets in cells that express crRNA arrays harboring independent sgRNAs (Zetsche et al., 2017). These tools have primarily been applied in order to characterize combinatorial gene interactions and to delete non-coding sequences. However, these strategies may also aid in studies where single gene knockouts are desired in each cell, as the simultaneous focusing of Cas9 to multiple sites within the target should elicit greater functional consequences.

## Design

Not all of the strategies outlined above have been experimentally validated, nor have they been integrated into a consolidated framework for constructing sgRNA expression vectors. We reasoned that a gain in sgRNA efficacy could be achieved by combining current selection methods with strategies to maximize the likelihood of functionally deleterious genomic scars. We developed an sgRNA selection algorithm that identifies putative targets based on predictive nucleotide combinations, the likelihood of an FSM, and whether the target lies in a functional domain. For effector delivery, we have developed a system that allows for the simultaneous expression of two independent sgRNAs from each construct. With the goal of expressing two guides for a single target in each construct, we have developed a computational algorithm that optimizes the likelihood of synergistic deleterious effects. These methods have been validated through a reanalysis of pre-existing data and by carrying out comparative multiplexed CRISPR screens. We have predicted construct designs for all protein-coding human genes and made these available via a web portal (http://croatan.hannonlab.org/).

## Results

### gRNA Selection Strategy

Two datasets of sgRNA efficacy have been used to develop existing selection algorithms. Doench et al. (2014) assessed the potency of sgRNAs in libraries that tiled cell surface proteins. There the abundance of integrated sgRNAs in FACS-isolated, target-negative cells was used as a measure of effector strength. Chari et al. (2015) infected cells with scrambled Cas9 targets and then transfected the same cells with corresponding sgRNAs. Here target mutation rates were the readout for efficiency. We developed a random-forest-based sgRNA prediction tool using these two datasets for training. For each dataset, ten random forests were trained to separate potent and weak guides, which were preclassified based upon a top- and bottom-40% efficacy cutoff, respectively. All 3mers in the region spanning four nucleotides upstream and six nucleotides downstream of the sgRNA binding site were used as input. The ten random forests were trained using incrementally increasing penalties for false-positive predictions. Thus, those trained with higher values were more stringent in assigning potency to a target. When analyzing new sgRNAs, sequences receive scores equal to the highest stringency level they pass in both random forest sets. This scoring system was applied to sgRNAs in the Doench tiling set that were withheld during training, and a significant difference in efficacy is observed when comparing sgRNAs that pass versus those that fail the minimum-stringency threshold (Figure S1A, rank-sum p value < 0.01). Beyond this, increments in prediction values are not matched with significant efficiency gains, although scores do correlate with potency globally.

**Figure 1:**
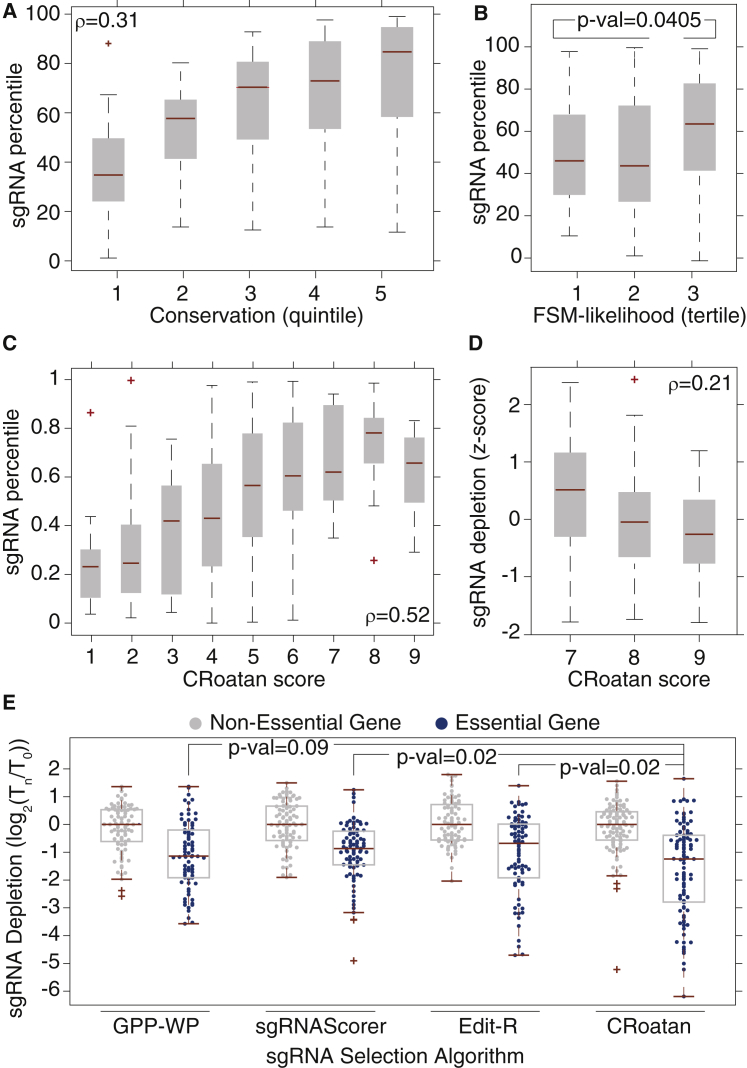
CRoatan, an Algorithm for Identifying Potent sgRNAs (A) The potency of sgRNAs analyzed in Doench et al. stratified by conservation score (calculated as described in Figure S1B, ρ = 0.32). An sgRNA percentile is the percentile rank of an sgRNA relative to all other effectors targeting the same gene. This plot, as all others in the figure, was generated with the MATLAB boxplot function using default parameters. The edges of the box are the 25^th^ and 75^th^ percentiles. The error bars extend to the values q3 + w(q3 − q1) and q1 − w(q3 − q1), where w is 1.5 and q1 and q3 are the 25^th^ and 75^th^ percentiles. (B) Efficacy percentiles of the sgRNAs analyzed in Doench et al. when stratified by the likelihood of frameshift mutations (FSM likelihood) at the corresponding target site (rank-sum p value = 0.0405 for tertile 3 versus tertile 1 and 2 sgRNAs). (C) Efficacy percentiles of the sgRNAs analyzed in Doench et al. when stratified by the consolidated CRoatan algorithm (ρ = 0.52). (D) *Z*-score-normalized depletion rates of EG-sgRNAs when stratified by CRoatan score (ρ = 0.21). Depletion rates were calculated as the average log ratio in screens carried out in A-375 and K-562 cells. (E) Depletion rates of NEG- and EG-targeting sgRNAs in screens corresponding to those described in (D). sgRNA libraries were designed using the GPP-WP, sgRNAScorer, and Edit-R algorithms (rank-sum p value = 0.0942 for GPP-WP, 0.0209 for sgRNAScorer, and 0.0233 for Edit-R).

The advantage of focusing Cas9 to known functional protein domains has been previously recognized (Shi et al., 2015). However, as many genes lack well-defined domain information, this strategy is not easily applied to the construction of genome-scale sgRNA collections. As a surrogate, we used amino-acid conservation at the Cas9 cut-site to guide sgRNA selection. We assigned scores to targets based on the predicted deleterious effects of DSB-proximal amino acid substitutions, which were calculated using the protein variation effect analyzer (PROVEAN) algorithm (Figure S1B; Choi et al., 2012). A reanalysis of the Doench tiling set shows that, for sgRNAs that pass the minimum random forest stringency threshold, these scores are correlated with the probability of inducing a measurable phenotype (Figure 1A, Spearman correlation [ρ] = 0.32).

Others have demonstrated that repair at Cas9-induced DSBs is partially driven by HEJ (Figure S1C; Bae et al., 2014). Using deep-sequencing data of Cas9 targets, we developed a linear regression model to predict the likelihood of homology-guided repair resolutions based on the length, GC content, and distance to the DSB of the corresponding homologous loci. The overall likelihood of an FSM at a target is measured as the fraction of predicted resolution scores that correspond to FSMs (Figure S1D, ρ = 0.74). This is only relevant for targets where homologous repair is likely. Thus, a lower-limit cutoff equal to the median of likelihood sums for HEJ-guided resolutions at human CDS Cas9 targets is applied as well. A reanalysis of the Doench dataset demonstrates that, for sgRNAs that pass the minimum random forest stringency threshold, a gain in efficacy can be attained by selecting targets where there is >66% chance that an FSM will occur (Figure 1B, rank-sum p value < 0.05).

To consolidate these predictive component algorithms, we first group sgRNAs based on the stringency level they passed during random-forest analysis (groups A, B, and C, Figure S1E). Within each group, sgRNAs are ranked based on their passing conservation and FSM-likelihood threshold tests. The median score of all human CDS Cas9 sites is the lower-limit threshold for conservation. We set a threshold of 66% to qualify sgRNAs as being likely to induce an FSM. sgRNAs in group A are given a score between one and three based on their passing zero, one, or two of the conservation and FSM-likelihood tests. With these same tests, sgRNAs in groups B and C are assigned scores between four and six and between seven and nine, respectively. A reanalysis of the Doench tiling set with this algorithm, which we call CRoatan, demonstrates that scores correlate strongly with potency (Figure 1C, ρ = 0.52). When CRoatan was applied to identify ten sgRNAs for each protein-coding gene in the refseq annotation, the algorithm could identify high-scoring sgRNAs for each target (Figure S1F).

To evaluate CRoatan empirically, we constructed four CRISPR libraries whose output would inform on the quality of the tool. Each library was composed of 200 sgRNAs targeting 20 essential and 20 nonessential genes (EG and NEG, respectively; five sgRNAs per gene). EGs were identified in a summary analysis of independent shRNA screens, and olfactory-receptor genes served as NEGs (Marcotte et al., 2012). For each library, a different sgRNA selection tool was used to define inclusion: gene perturbation platform (GPP; Doench et al., 2014), sgRNAScorer (Chari et al., 2015), Edit-R (Dharmacon), and CRoatan. Libraries were cloned into a lentiviral backbone where human U6 drives sgRNA expression and where a zsGreen-P2A-Puromycin bicistronic transcript is expressed from the spleen focus-forming virus promoter (SFFV). Libraries were packaged and infected into A-375 melanoma and K-562 leukemia cells, and following selection with puromycin, the cells were passaged for ∼12 doublings. Normalized log ratios were then calculated based on construct abundances in the infected and final cell populations (Knott et al., 2014).

To assess the effectiveness of our effector selection strategies, we calculated gene-normalized depletion scores for all EG-sgRNAs in the CRoatan library. We could not test the initial grouping strategy, as all sgRNAs were group C members (Figure S1E). The depletion rates of EG-sgRNAs were correlated with CRoatan score (Figure 1D, ρ = 0.52). Depletion rates were not found to correlate with conservation or FSM-likelihood scores alone. When EG-sgRNA depletion rates were compared among the four libraries, CRoatan sgRNAs were found to be significantly more reduced in representation than those identified with the sgRNAScorer and Edit-R tools (Figure 1E, rank-sum p value < 0.05). CRoatan EG-sgRNAs were more depleted than those identified with the GPP algorithm; however, this difference was not statistically significant (rank-sum p value > 0.05).

### Dual-sgRNA Expression Constructs

**Figure 2:**
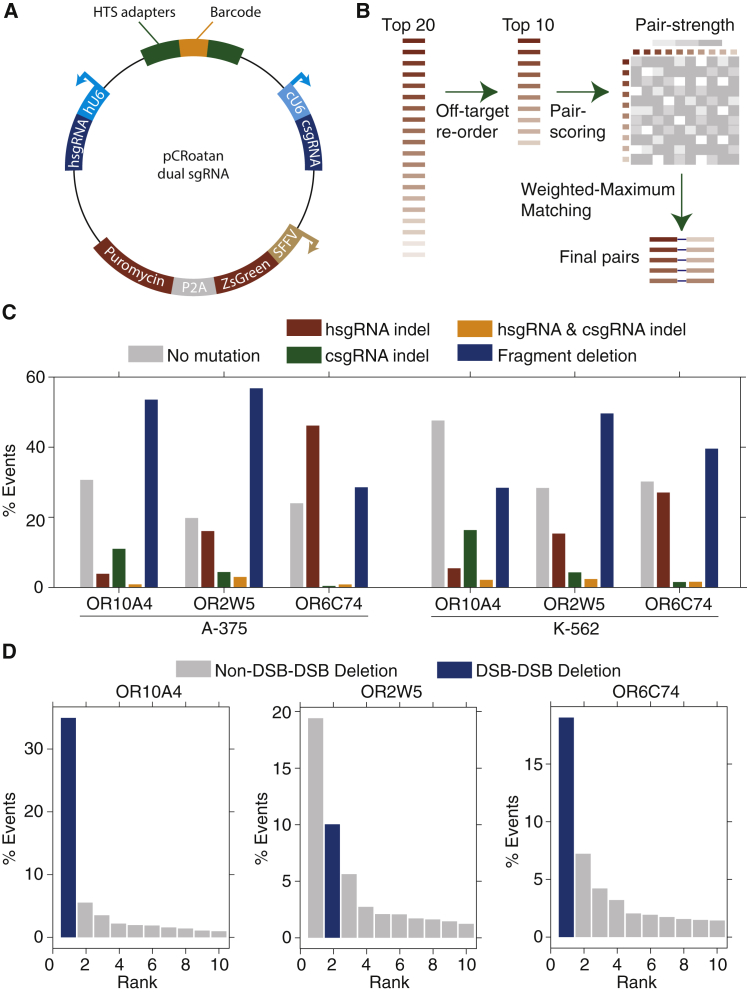
Simultaneous Targeting with Multiple sgRNAs Results in Predictable Genomic Scars (A) Schematic map of the lentiviral, dual-sgRNA expression vector with relevant features highlighted. hU6, human U6 promoter; cU6, chicken U6 promoter; hsgRNA, human U6-promoter-driven sgRNA; csgRNA, human U6-promoter-driven sgRNA; HTS, high-throughput sequencing adapters; SFFV, spleen focus-forming virus promoter. (B) sgRNA pairing algorithm used to design five targeting constructs for a gene. The top 20 sgRNAs for each gene are filtered to a set of 10 to reduce the probability of off-targeting effects. Pairs within these 10 are then scored using the set of heuristics defined in Figure S2. The resultant pairing matrix is then used as input for a maximum-weighted matching algorithm to define a final set of 5 sgRNA pairs. (C) Paired-end sequencing analysis of genomic scars left after dual-CRoatan NEG-targeting constructs have been infected into A-375 and K-562 cells. hsgRNA and csgRNA indels are where only one of the two targeted regions shows mutational burden in an HTS fragment. hsgRNA and csgRNA indel counts represent cases where both targets have indels, and fragment deletions are where the region between the two targets is deleted. (D) Analysis of the genomic scars described in (C) that correspond to fragment deletions between two sgRNA target sites. The top ten most frequent deletions are shown with their corresponding rate of occurrence, as measured by their average frequency in infected A-375 and K-562 cells. Scars that result from exact deletion of the double-strand-break-flanked fragment are annotated as DSB-DSB deletions. Scars where, in addition to the fragment deletion, other bases are inserted or deleted are annotated as non-DSB-DSB deletions.

We reasoned that a higher frequency of deleterious mutations could be inflicted by simultaneously focusing multiple independent sgRNAs to each gene target. Toward this end, we constructed a lentiviral vector harboring two divergent U6 promoters, where the 5′ promoter was human and the 3′ promoter was chicken (Figure 2A, hU6 and cU6, respectively). These were chosen to reduce the probability that recombination would eliminate critical elements of the cassette. Between the promoters is an identification barcode, which is bordered by Illumina adapters, for sequencing-based quantification of construct abundances. The vector also harbors a bicistronic zsGreen-P2A-Puromycin transcript that is expressed from SFFV.

We designed an algorithm to pair sgRNAs for a target within the dual-U6 vector to maximize the probability of synergistic deleterious effects. The algorithm receives as input ten sgRNAs, which have been extracted from the top-20 CRoatan-scoring effectors, after they have been reranked to reflect off-target likelihoods (Figure 2B; Knott et al., 2014). A 10 × 10 pairwise score matrix is then calculated using a heuristic scoring algorithm (Figure S2A). sgRNAs with overlapping targets are not considered for pairing. To ensure that each construct harbors at least one potent effector, the algorithm increments the score of sgRNA pairs with unbalanced CRoatan scores. sgRNA pairs are also increased in their scores if they target the same exon or two exons that contribute to a common set of isoforms. Finally, we predicted that DSB-DSB blunt-end joining would be the predominant repair resolution in cases where simultaneous cleavage events caused the target-flanked region to be deleted. Thus, the score is also increased for each pair whose deletion fragment length corresponds to an FSM. After the sgRNA pairs have been scored, a weighted maximum matching algorithm is applied to identify the coupling with the highest sum of pair scores.

To test our library assembly strategy, we cloned dual-CRoatan constructs for three olfactory receptor genes (OR10A4, OR2W5, and OR6C74) and infected A-375 and K-562 cells with these. These sgRNA pairs were chosen for the short distance between their corresponding targets, which allows simultaneous analysis of both sites with Illumina paired-end sequencing. We profiled the genomic scars that had been left after infection and found that high rates of mutation existed for all sgRNA pairs (Figure 2C). Fragment deletion between the two targets was the predominant scar. A deeper analysis revealed that, in these cases, the most commonly observed resolution was the predicted blunt-end joining of the two DSBs (Figures 2D and S2B).

### High-Throughput Analysis of Library Efficacy

To evaluate our strategy more broadly, we designed a combinatorial CRISPR screening library whose output would inform on the contributions that the CRoatan algorithm, as well as the dual-sgRNA expression system, made to reagent efficacy. The library was composed of 100 sgRNAs targeting 20 EGs and 20 NEGs. sgRNAs were cloned into both the hU6 and cU6 positions, which resulted in a final library harboring 10,000 sgRNA pairs. The constructs were screened in A-375 cells, and these experiments were processed in the same manner as those experiments described in Figures 1D and 1E.

To assess the impact of the CRoatan algorithm constituents, we calculated gene-normalized depletion scores for constructs harboring one EG-sgRNA, as depletion rates could be attributed directly to the efficacy of this effector for these constructs. In contrast to the single-sgRNA CRoatan screen described in Figures 1D and 1E, here depletion rates were significantly greater for EG-sgRNAs that passed the conservation and FSM-likelihood thresholds, indicating that these two strategies contributed positively (Figures S3A and S3B, Friedman p value < 0.05). We reason that this correlation was observable here, and not in the initial CRoatan screen, because for each EG-sgRNA, the score was calculated as the average depletion rate of the 100 constructs in which it was paired with an NEG-sgRNA. Also, as was the case for the initial CRoatan screen, depletion rates correlated positively with CRoatan score (Figure S3C, Friedman p value < 0.01).

**Figure 3:**
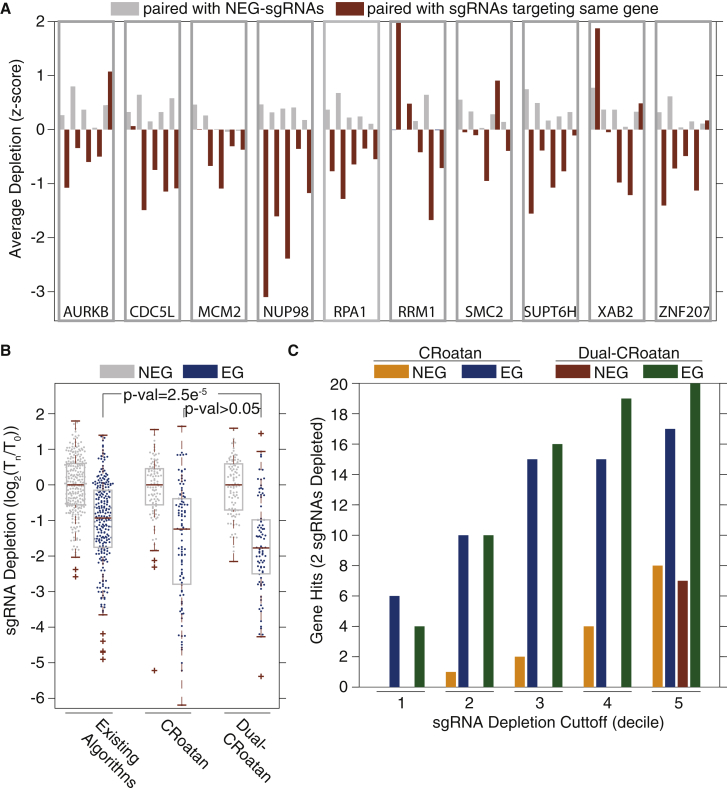
Dual-CRoatan Constructs Provide Superior CRISPR-Based Gene Targeting (A) Average depletion rates for each EG-sgRNA when it is paired with NEG-sgRNAs (gray) and when it is paired with sgRNAs targeting the same EG (brown). sgRNAs are grouped based on the gene target; rank-sum p value = 0.0006. (B) Depletion rates of NEG- and EG-targeting CRISPR constructs in negative-selection screens. Shown are the consolidated depletion rates for single-sgRNA constructs selected using pre-existing tools (GPP, sgRNAScorer, or Edit-R algorithms) as well as the rates for CRoatan single-sgRNA constructs and CRoatan dual-sgRNA constructs (dual-CRoatan, rank-sum p value = 2.5e^-5^ for existing algorithms and p value > 0.05 for single-CRoatan constructs). Depletion rates were calculated as the average log ratio in screens carried out in A-375 and K-562 cells. This plot was generated with the MATLAB boxplot function using default parameters. The edges of the box are the 25^th^ and 75^th^ percentiles. The error bars extend to the values q3 + w(q3 − q1) and q1 − w(q3 − q1), where w is 1.5 and q1 and q3 are the 25^th^ and 75^th^ percentiles. (C) Gene-level analysis of CRoatan and CRoatan dual-sgRNA construct depletion rates. Using the average depletion rate for each construct in A-375 and K-562 cells, gene “hits” were calculated using a series of stringencies (top 10%, 20%, 30%, 40%, and 50% most-depleted sgRNAs). For a gene to be called a hit at a given stringency, a minimum of two constructs need to be depleted beyond the stringency level.

A significant increase in depletion levels was also observed when constructs harboring two EG-sgRNAs were compared to those harboring one or zero EG-RNAs (Figure S3D, rank-sum p value < 0.01). This was also evident at the individual sgRNA level. For each EG-sgRNA, we calculated the mean depletion rate of constructs where it was paired with an NEG-sgRNA and also where it was paired with one of the other four sgRNAs that target the same gene. Nearly all of the EG-sgRNAs elicited a more robust phenotype when they were paired with other sgRNAs targeting the same gene (Figure 3A, rank-sum p value < 0.001).

As a final test of our consolidated strategy, we constructed a CRISPR library using the CRoatan algorithm and the pairing principles outlined in Figures 2A, 2B, and S2A (dual-CRoatan). Each construct in the library harbors two sgRNAs that together target one of the 10 EGs or 10 NEGs described in Figures 1D and 1E for multiplexed mutagenesis. The library was screened and analyzed as was described for these earlier experiments. The EG-targeting dual-CRoatan constructs had significantly higher depletion rates than the single-sgRNA constructs. This was true when all sgRNAs identified with existing algorithms were considered together and also when sgRNAs identified with the GPP, sgRNAScorer, and Edit-R algorithms were considered separately (rank-sum p values = 2.4e^-5^, 0.005, 3.9e^-5^, and 0.002, respectively). EG-targeting constructs in the dual-CRoatan library were more depleted than their counterparts in the CRoatan library; however, this difference was deemed statistically insignificant (rank-sum p value > 0.05). Finally, we analyzed the CRoatan and dual-CRoatan screens to identify gene-level “hits.” Using a two-construct minimum threshold to identify a gene as depleted, we calculated false-positive and true-positive rates at a series of construct depletion cutoffs. This analysis demonstrated the superiority of the dual-CRoatan library in terms of both sensitivity and specificity (Figure 3C).

## Discussion

The CRISPR-Cas9 system has been applied to a variety of molecular manipulations, with the most common being perturbation of gene function in mammalian cells. This can be achieved by inducing mutations in target gene coding sequences or by focusing transcriptional regulators to gene promoters. Others have demonstrated, through a set of parallel loss-of-function screens, that mutagenesis is more effective at ablating gene function. Here we have combined machine-learning and sgRNA-expression strategies to create CRISPR constructs that maximize the likelihood of mutation-based functional silencing. Through a set of parallel genetic screens, we have demonstrated that these reagents are significantly more efficacious than other available tools. Based upon these results, we have assembled a sequence-verified collection of CRISPR constructs using these design principles.

We have demonstrated that a significant gain in efficacy is attained when two independent sgRNAs simultaneously focus Cas9 to the target gene. Thus, we have designed the library such that two sgRNAs with high prediction scores are expressed from each construct (Figure S3E). An added benefit of this strategy is that constructs can be easily manipulated to target gene pairs to interrogate synthetic interactions. This feature will be particularly useful for identifying parallel or related molecular pathways with combinatorial screens. Another feature of the toolkit is the availability of individual sequence-verified constructs, which allows large-scale screens to be carried out in an arrayed format.

Overall, we hope that this toolkit will be of benefit to the scientific community, as it will allow individual and combinatorial gene knockouts to be carried out on a large scale in both multiplexed and arrayed formats. The library design includes five constructs for each protein coding human Refseq gene. At present, the library is comprised of ∼50,000 sequence-verified constructs; the goal is to complete the collection at five constructs per ∼20,000 predicted genes.

## Limitations

At the date of publication, half of the ∼100,000 construct designs in the human library had been sequence verified and included in the physical resource. Thus, there is poor coverage, in terms of targeting molecules, for a subset of genes. Current coverage statistics are reported on the following web portal: http://croatan.hannonlab.org.

## STAR★Methods

### Key Resources Table

**Table:** 

REAGENT or RESOURCE	SOURCE	IDENTIFIER
**Deposited Data**		

Raw and analyzed data	This paper	GEO: GSE97434
Human reference genome NCBI build 37, GRCh37	Genome Reference Consortium	http://www.ncbi.nlm.nih.gov/projects/genome/assembly/grc/human/

**Experimental Models: Cell Lines**		

A-375	ATCC	CRL-1619
A-375-Cas9	This paper	
K562-Cas9	Gift by the Vakoc Laboratory (CSHL)	

**Oligonucleotides**		

Primers used to generate sgRNAs libraries, see Table S1	This paper	N/A
Sequenced included in the DNA chip used to clone combinatorial sgRNA libraries, see Table S2	This paper	N/A
Primers used to amplify sgRNAs from gDNA and sequence targeted loci, see Table S3	This paper	N/A

**Recombinant DNA**		

pCRoatan-dualSgRNA	This paper	N/A
pCRoatan-singleSgRNA	This paper	N/A
pCRoatan-dualPromoter	This paper	N/A

**Software and Algorithms**		

Bowtie	Langmead et al., 2009	RRID: SCR_005476
Bwa	Li and Durbin, 2009	RRID:SCR_010910
**Other**		

Resource website for the paper	This paper	http://croatan.hannonlab.org/
CRoatan dual-sgRNA cloning protocol	This paper	Methods S1

### Contact for Reagent and Resource Sharing

Further information and requests for resources and reagents should be directed to and will be fulfilled by the Lead Contact, Gregory Hannon (greg.hannon@cruk.cam.ac.uk).

### Experimental Model and Subject Details

#### Cell Lines

CRISPR/Cas9 screens were performed in melanoma A-375 (ATCC CRL-1619, female) and chronic myelogenous leukemia K-562 (ATCC CCL-243, female) cell lines. A-375 were grown at 37C in DMEM, supplemented with 10% FBS and penicillin/streptomycin. K-562 were grown at 37C in RPMI1640 supplemented with 10% FBS and penicillin/streptomycin. The 293FT cell line (Thermo-Fischer) was grown at 37C in DMEM supplemented with 10% FBS and penicillin/streptomycin.

A-375 cells were infected at low MOI by virus produced using lentiCas9-Blast (Addgene #52962) (Sanjana et al., 2014) and selected using blasticidin (10 μg/mL). Following 10 days of selection, single cells were sorted using the FACSAria IIU cell sorter (BD Biosciences) into 96-well plates. 10 A-375-Cas9 clones were tested for Cas9 functionality by infection with a vector expressing ZsGreen and an sgRNA targeting ZsGreen. Knockout efficiency was estimated by flow cytometry after 14 days. One of the A-375-Cas9 clonal lines exhibiting more than 50% knockout of ZsGreen in this assay was selected for further experiments. The K-562 clonal cell line expressing Cas9 was kindly gifted by Dr. Vakoc (Cold Spring Harbor Laboratory).

### Method Details

#### Random Forest Training and Scoring

Ten random forests were constructed for each of the Doench et al. and Chari et al. datasets. For each data type, sgRNAs in the top- and bottom-40^th^ percentile for each gene were classified as potent and weak, respectively. The 10 forests were trained using the MATLAB treeBagger package (1000 trees per forest). Forests were trained using incrementally increasing penalties for false-positive classifications (0.2, 0.4, 0.6, 0.8, 1, 1.2, 1.4, 1.6, 1.8, 2). During training forests are constructed using the 28 overlapping 3mers of each target as features, and the class of the target (potent or weak) as the output.

When a new target is being scored, it is decomposed into 28 3mers, and these are given to each of the 20 forests (10 corresponding to the Doench et al. data and 10 to the Chari et al. data) as input. The target is then assigned a value between 0 and 10 corresponding to the highest stringency forest it was assigned as potent by. For example, if a target was called potent by a Doench forest that was trained with a penalty of 1.2 (6^th^ lowest) and a Chari forest trained with a penalty of 1 (5^th^ lowest), the target would receive a score of 5. The data presented in Figures S1A and 1C were calculated using out-of-bag random forest predictions with default MATLAB parameters.

#### sgRNA-Pair Scoring

For each gene, all pairwise scores were calculated for the top 10 CRoatan scoring sgRNAs. All sgRNA pairs begin with a score of 0. Overlapping pairs are assigned a final score of 0. Pairs that are less than 10kb apart with DSB-DSB distances that are not divisible by 3 are assigned a score of 2.5 if they target the same transcripts. Scores are incremented by 1 if pairs have imbalanced CRoatan scores (one less than 7 and one greater than 7). This scoring matrix is then given as input to the maximum weighted matching algorithm (MATLAB maxWeightMatching).

#### sgRNA Library Construction

For single sgRNA libraries, sgRNA sequences were predicted using existing algorithms (Edit-R, sgRNAScorer, GPP web portal and CRoatan) and oligonucleotides containing these sequences were ordered from Integrated DNA Technologies (IDT, Table S1). These molecules were amplified by PCR (forward primer (FP): TTACCGTAACTTGAAAGTATTTCGATTTCTTGGCTTTATATATCTTGTGGAAAGGACGAAACACCG, reverse primer (RP): GGACTAGCCTTATTTTAACTTGCTATTTCTAGCTCTAAAAC) and cloned by Gibson assembly into a 3^rd^ generation lentiviral vector harboring a U6 promoter, an sgRNA backbone, and a ZsGreen-P2A-Puromycrin^R^ transcript driven by a spleen focus-forming virus promoter (pCRoatan-singleSgRNA).

For dual sgRNA libraries, sgRNA sequences were predicted using CRoatan. Primers containing these sequences were ordered from IDT (Table S1) and used to amplify a hU6-EM7-Zeocin^R^-cU6 cassette (pCRoatan-dualPromoter). The amplicon was digested with BbsI (NEB) and ligated into a 3^rd^ generation lentiviral vector (pCRoatan-dualSgRNA) previously digested with BsmBI (ThermoFischer).

Combinatorial sgRNA libraries were built using DNA chips (CustomArray, Inc.) containing 10K molecules harboring a barcode and two flanking sgRNA sequences (Table S2). Chips were amplified by 5 separate 18-cycle PCRs to ensure high-complexity end product. The amplicons were first cloned by ligation into an intermediate cloning vector (pCR-BluntII TOPO based) using SpeI (NEB) and ApaI (NEB). Subsequently, the hU6 and cU6 promoters driving the sgRNAs were added to the vector. The hU6 promoter was amplified from lentiCrisprv2 (Addgene #52961) by PCR (FP: AGTACCGTCTCTGGTGTTTCGTCCTTTCCACAAG, RP: GTACCTACGCGTGAGGGCCTATTTCCCATGATTC), and cloned by ligation using the BsmBI (ThermoFischer) and MluI (NEB) restriction sites. The cU6 promoter (cU6-3, Kudo and Sutou, 2005) was amplified from a gBlock (IDT) by PCR (FP: ATCGATCTCGAGGCGCCGCCGCTCCTTCAGGCA, RP: TGATCCTGGTCTCACGACTAAGAGCATCGAGACTGC), and cloned by ligation using the BsaI (NEB) and XhoI (NEB) restriction sites. Following these three steps, the full sgRNA1-hU6-EM7-Zeocin^R^-Barcode-cU6-sgRNA2 cassette was digested from the intermediate cloning vector using BbsI and ligated in the lentiviral expression vector (pCRoatan-dualSgRNA) as described previously. All transformations were performed with Invitrogen’s MegaX DH10B T1 electro-competent cells using a Bio-Rad Gene Pulser Xcell and Bio-Rad Gene Pulser 1 mm cuvettes for electroporation. For each library, a minimum of 10 million successfully transformed cells were obtained.

#### sgRNA Library Screening

sgRNA libraries were packaged using the 293FT cell line (Thermo Fischer). Cells were co-transfected with library vector (60 μg), pMDL (12.5 μg), CMV-Rev (6.5 μg) and VSV-G (9 μg) by calcium phosphate transfection. The media was replaced at 14h and virus was collected at 36h and filtered using a 0.45 μM syringe filter (Millex®-HV, EMD Millipore). Viral infections were performed at an MOI of 0.3 to ensure a maximum of one sgRNA integration per cell. sgRNA representation in the infected population was maintained at a minimum of 1000 infected cells per sgRNA at each passage. All screens were performed in triplicates. Two days after infection, cells were collected for a reference time point. After ∼12 doublings, cells were harvested for a final time point. Infected cells were selected using Puromycin (1 μg/mL) after the initial time point and throughout the screen.

#### CRISPR/Cas9 Library Processing and Analysis

Following cell harvests, DNA was extracted using the QIAGEN QIAamp DNA Blood Midi kit. For each sample, sgRNA molecules or barcodes identifying sgRNA pairs were extracted from the genomic DNA in 24 separate 30-cycle PCR reactions in which 2 μg of DNA input was included. Illumina adapters were included in the PCR primers (Table S3). Libraries were sequenced using custom read one primers on the Illumina MiSeq or HiSeq platforms. Following sequencing, reads were trimmed to a length of 20bp and construct counts were extracted using the bowtie algorithm (Langmead et al., 2009). Constructs were then filtered based on a minimum read-count threshold of 50 in the reference sample. Corresponding log-fold change values were then calculated by dividing the abundance after twelve doublings by the abundance at the reference time point, two days after infection (Knott et al., 2014).

#### Dual-sgRNA Genomic Scar Analysis

200,000 A-375-Cas9 and K-562-Cas9 cells were transduced with CRoatan constructs targeting 3 different olfactory receptor genes. Following selection with Puromycin cells were grown for ∼12 doublings and then harvested for analysis. DNA was extracted using the QIAGEN QIAamp DNA Blood Midi kit. The target region, including 50bp upstream and downstream of both sgRNA target sites was amplified by PCR, in 16 25-cycle PCR reactions in which 500ng of DNA input was included (Table S3). Following purification using the QIAquick PCR Purification Kit, Illumina adapters were added via PCR and samples were processed on the Illumina MiSeq platform using paired-end reads of 200bp to cover both sgRNA target sites. Reads were mapped to the relevant genomic region using the bwa mem algorithm and cut types were analyzed and counted using the CIGAR string of the alignment (Li and Durbin, 2009).

### Quantification and Statistical Analysis

Statistical parameters such as definition of center, error bars and significance are reported in the main text, figures and figure legends. Data are judged to be significant when p < 0.05 by the rank-sum test or the Friedman test. Statistical significance analysis was performed in MATLAB using the freidman and ranksum functions.

### Data and Software Availability

All raw and processed data have been deposited in the National Center for Biotechnology Information Gene Expression Omnibus under accession number GSE97434. All code will be made available for non-commercial use upon request.

### Additional Resources

#### Detailed Protocol

A detailed protocol describing the cloning of pairs of sgRNAs in the pCRoatan-dualSgRNA expression vector is provided in the Methods S1.

#### Online Resource

Detailed cloning protocols, plasmid maps and construct designs for all protein coding human genes are available via a web portal: http://croatan.hannonlab.org.

## Author Contributions

N.E., S.R.V.K., and G.J.H. designed the experiments and wrote the manuscript. S.R.V.K. designed the prediction algorithm and analyzed the CRISPR screens. N.E. performed all experiments. S.R.V.K. and G.J.H. supervised all experiments.

## Supplemental Information

Document S1. Figures S1–S3

Table S1. sgRNA Library Construction, Related to Star Methods Oligonucleotide sequences used to generate the sgRNA libraries used in the depletion screens.

Table S2. sgRNA Library Construction, Related to Star Methods Oligonucleotide sequences used to create the dual-sgRNA combinatorial library.

Table S3. CRISPR/Cas9 Library Processing and Analysis, Related to Star Methods List of primers used to PCR-amplify and sequence screen sgRNAs and the loci targeted by dual-sgRNA constructs in one-by-one experiments.

Methods S1. Detailed Protocol, Related to Star Methods Detailed protocol describing the cloning of pairs of sgRNAs in the pCRoatan-dualSgRNA expression vector.

Document S2. Article plus Supplemental Information

## References

[bib1] Bae S., Kweon J., Kim H.S., Kim J.S. (2014). Microhomology-based choice of Cas9 nuclease target sites. Nat. Methods.

[bib2] Carpenter A.E., Sabatini D.M. (2004). Systematic genome-wide screens of gene function. Nat. Rev. Genet..

[bib3] Chari R., Mali P., Moosburner M., Church G.M. (2015). Unraveling CRISPR-Cas9 genome engineering parameters via a library-on-library approach. Nat. Methods.

[bib4] Choi Y., Sims G.E., Murphy S., Miller J.R., Chan A.P. (2012). Predicting the functional effect of amino acid substitutions and indels. PLoS ONE.

[bib5] Doench J.G., Hartenian E., Graham D.B., Tothova Z., Hegde M., Smith I., Sullender M., Ebert B.L., Xavier R.J., Root D.E. (2014). Rational design of highly active sgRNAs for CRISPR-Cas9-mediated gene inactivation. Nat. Biotechnol..

[bib6] Doench J.G., Fusi N., Sullender M., Hegde M., Vaimberg E.W., Donovan K.F., Smith I., Tothova Z., Wilen C., Orchard R. (2016). Optimized sgRNA design to maximize activity and minimize off-target effects of CRISPR-Cas9. Nat. Biotechnol..

[bib7] Fellmann C., Zuber J., McJunkin K., Chang K., Malone C.D., Dickins R.A., Xu Q., Hengartner M.O., Elledge S.J., Hannon G.J., Lowe S.W. (2011). Functional identification of optimized RNAi triggers using a massively parallel sensor assay. Mol. Cell.

[bib8] Knott S.R., Maceli A.R., Erard N., Chang K., Marran K., Zhou X., Gordon A., El Demerdash O., Wagenblast E., Kim S. (2014). A computational algorithm to predict shRNA potency. Mol. Cell.

[bib9] Kudo T., Sutou S. (2005). Usage of putative chicken U6 promoters for vector-based RNA interference. J. Reprod. Dev..

[bib10] Langmead B., Trapnell C., Pop M., Salzberg S.L. (2009). Ultrafast and memory-efficient alignment of short DNA sequences to the human genome. Genome Biol..

[bib11] Li H., Durbin R. (2009). Fast and accurate short read alignment with Burrows-Wheeler transform. Bioinformatics.

[bib12] Marcotte R., Brown K.R., Suarez F., Sayad A., Karamboulas K., Krzyzanowski P.M., Sircoulomb F., Medrano M., Fedyshyn Y., Koh J.L. (2012). Essential gene profiles in breast, pancreatic, and ovarian cancer cells. Cancer Discov..

[bib17] Pelossof R., Fairchild L., Huang C.H., Widmer C., Sreedharan V.T., Sinha N., Lai D.Y., Guan Y., Premsrirut P.K., Tschaharganeh D.F. (2017). Prediction of potent shRNAs with a sequential classification algorithm. Nat. Biotechnol..

[bib13] Sanjana N.E., Shalem O., Zhang F. (2014). Improved vectors and genome-wide libraries for CRISPR screening. Nat. Methods.

[bib14] Shi J., Wang E., Milazzo J.P., Wang Z., Kinney J.B., Vakoc C.R. (2015). Discovery of cancer drug targets by CRISPR-Cas9 screening of protein domains. Nat. Biotechnol..

[bib15] Vidigal J.A., Ventura A. (2015). Rapid and efficient one-step generation of paired gRNA CRISPR-Cas9 libraries. Nat. Commun..

[bib16] Zetsche B., Heidenreich M., Mohanraju P., Fedorova I., Kneppers J., DeGennaro E.M., Winblad N., Choudhury S.R., Abudayyeh O.O., Gootenberg J.S. (2017). Multiplex gene editing by CRISPR-Cpf1 using a single crRNA array. Nat. Biotechnol..

