# Supplementary material for: A CRISPR Resource for Individual, Combinatorial, or Multiplexed Gene Knockout
Source: Mol Cell. 2017 Jul 20;67(2):348–354.e3. doi: 10.1016/j.molcel.2017.06.030 (PMC5526787; doi:10.1016/j.molcel.2017.06.030)

Molecular Cell, Volume *67*

Supplemental Information

A CRISPR Resource for Individual, Combinatorial, or Multiplexed Gene Knockout

Nicolas Erard, Simon R.V. Knott, and Gregory J. Hannon

Supplemental Figure Legends

Figure S1: Constituent CRoatan sub-algorithms, related to Figure 1.

(A) sgRNA percentiles of the sgRNAs analyzed in Doench et al. 2014, when stratified by the maximum stringency level of random forest they passed (rank-sum p-value = 0.032 for sgRNAs with scores 0 and 1, rank-sum p-value > 0.05 for all other increments). An sgRNA percentile is the percentile rank of an sgRNA relative to all other effectors targeting the same gene. This plot was generated with the Matlab Boxplot function using default parameters. The edges of the box are the 25th and 75th percentiles. The error bars extend to the values q3 + w(q3 − q1) and q1 − w(q3 − q1), where w is 1.5 and q1 and q3 are the 25th and 75th percentiles. (**B**) Strategy for assigning conservation scores to CRISPR targets using the PROVEAN predictions of double strand break (DSB)-flanking amino acids (AA) **(C)** Schematic representation of homologous and non-homologous end-joining (NHEJ and HEJ, respectively) repair resolution at Cas9-induced DSBs **(D)** Experimental vs. predicted rate of HEJ-induced frame-shift mutations (FSMs, ρ = 0.74) **(E)** Schematic of the consolidated CRoatan scoring algorithm. sgRNAs are initially grouped based on random forest score into group A, B and C. Each group is then re-ranked based on the conservation of the DSB-flanking AAs and the likelihood of an FSM at the target. **(F)** Representation of final CRoatan scores in the human sgRNA library (10 sgRNAs for each of the ~20,000 genes in the refseq protein-coding genome).

Figure S2: sgRNA pairing strategies to optimize synergistic deleterious effects, related to Figure 2.

(A) Schematic representation of the heuristics used to score sgRNA pairs prior to applying weighted-maximum-matching to identify final couples for inclusion in the dual-sgRNA vector. Additive features include imbalanced CRoatan scores, co-targeting of exons included in the same transcript and a distance between the two DSB sites that is not divisible by 3. Subtractive features include overlapping targets, targets within exons of different transcripts and distance between the two DSB sites being divisible by 3. **(B)** Analysis of the genomic scars described in Figure 2C, that correspond to fragment deletions between two sgRNA target sites. The top-10 most frequent deletions are shown with their corresponding rate of occurrence.

Figure S3: Assessment of the strategies employed in the dual-CRoatan design process, related to Figure 3.

(A) Gene-normalized depletion rates for constructs harboring one EG-sgRNA, stratified by the conservation status of that effector (friedman p-value = 0.03). This plot, as well as panels **B**, **C** and **D**, were generated with the Matlab Boxplot function using default parameters. The edges of the box are the 25th and 75th percentiles. The error bars extend to the values q3 + w(q3 − q1) and q1 − w(q3 − q1), where w is 1.5 and q1 and q3 are the 25th and 75th percentiles. **(B)** Gene-normalized fold-changes for the same constructs, stratified by FSM-likelihood (friedman p-value = 0.02) **(C)** Gene-normalized depletion rates for the same constructs, stratified by CRoatan score (friedman p-value = 0.03 and 0.02 for sgRNAs with CRoatan scores of 7 and 8, and 8 and 9, respectively). **(D)** Depletion rates for constructs harboring 0, 1 or 2 EG-sgRNAs (rank-sum p-value < 0.01 for constructs harboring 0 vs 1 and 1 vs 2 EG-sgRNAs) **(E)** Heatmap representation of CRoatan scores for sgRNA pairs contained in the human CRoatan library (5 constructs per gene, ~20,000 genes).

Erard et al. 2017, Figure S1

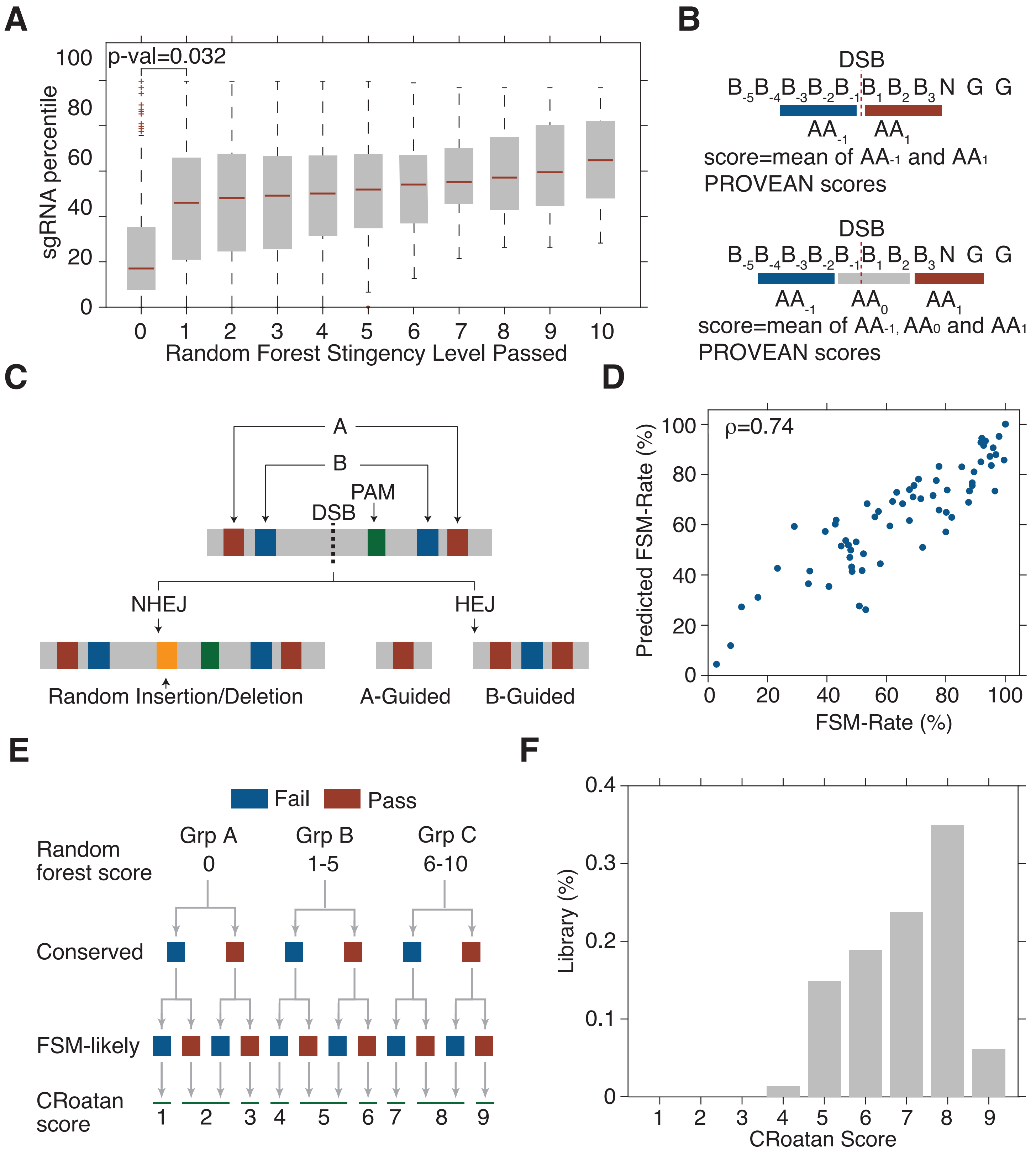

Erard et al. 2017, Figure S2

A

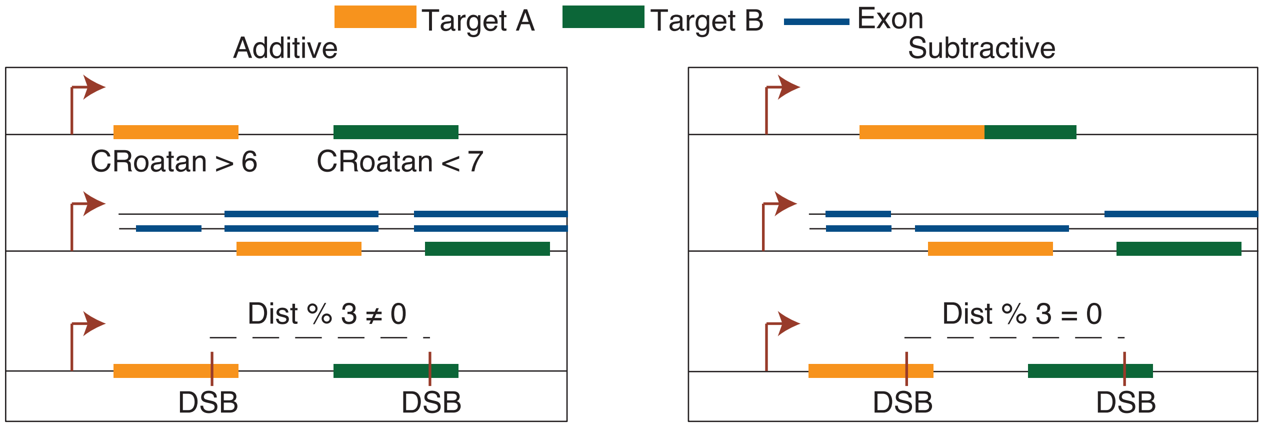

B

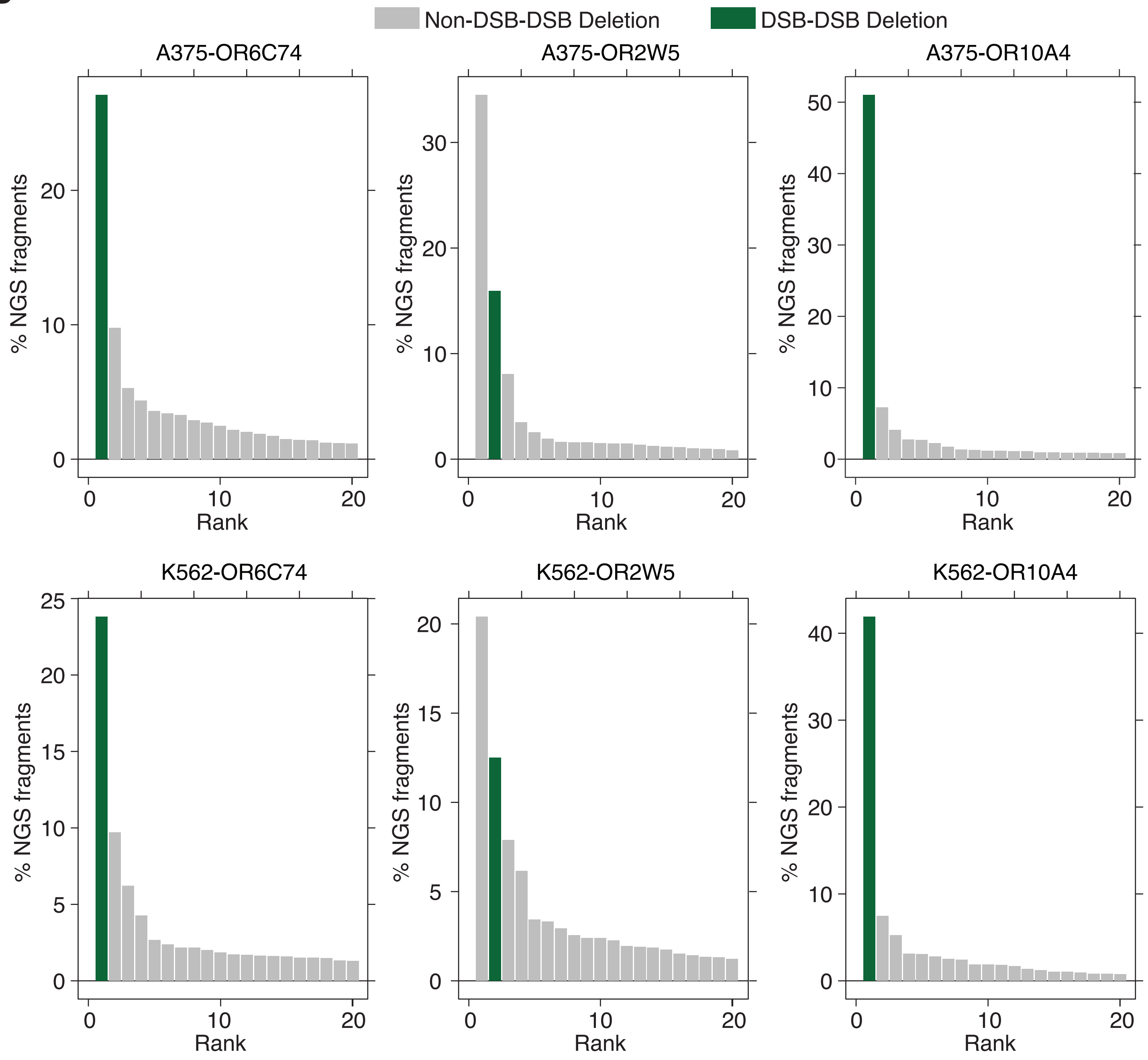

Erard et al. 2017, Figure S3

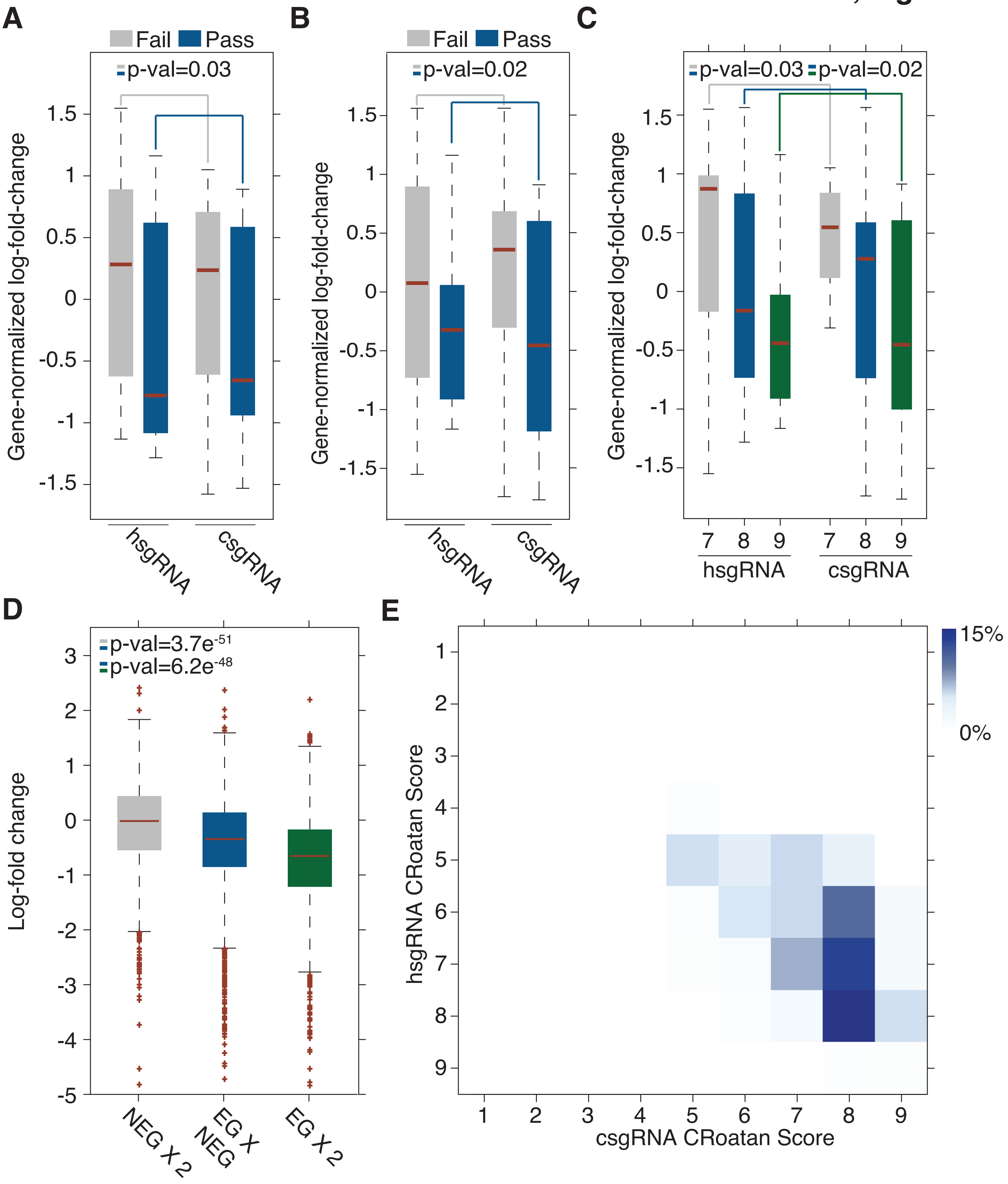

Supplement: Document S1. Figures S1–S3 [file mmc1.pdf]
