# Supplementary material for: A CRISPR Resource for Individual, Combinatorial, or Multiplexed Gene Knockout
Source: Mol Cell. 2017 Jul 20;67(2):348–354.e3. doi: 10.1016/j.molcel.2017.06.030 (PMC5526787; doi:10.1016/j.molcel.2017.06.030)
Supplement: Methods S1. Detailed Protocol, Related to Star Methods — Detailed protocol describing the cloning of pairs of sgRNAs in the pCRoatan-dualSgRNA expression vector. [file mmc5.pdf]

CRoatan dual-sgRNA cloning protocol

To clone pairs of sgRNAs into the pCRoatan dual-sgRNA expressing plasmid, synthetize two oligos of the following sequence:

sgRNA1-oligo	AGCGGAAGACGCTCTAAAACNNNNNNNNNNNNNNNNNNNNCGGTGTTTCGTCCTTTCCAC
sgRNA2-oligo	GGCAGAAGACTAAAACNNNNNNNNNNNNNNNNNNNNCCGACTAAGAGCATCGAGACTGC

In both oligos, the Ns are the reverse-complement of the 20bp target sequence. Standard desalted oligos can be used, resuspend the oligos to a final concentration of 100uM in water.

Step 1. Digest 4ug of pCRoatan-dualSgRNA plasmid with BsmbI for 2 hours at 37C:

4 ug	pCRoatan-dualSgRNA
5 ul	10x Buffer Tango (Thermo Fischer)
5 ul	10 mM DTT
2 ul	BsmBI (Thermo Fischer)
X ul	H_2O
50 ul	Total volume

Step 2. Gel purify the digested plasmid using a QIAquick Gel Extraction Kit. A ~1kb filler should visible on the gel if the digestion was successful. Extract the top, ~7.5kb band.

Step 3. Amplify the dual-promoters using the two oligos as primers:

1 ul	100 uM sgRNA1-oligo
1 ul	100 uM sgRNA2-oligo
1 ul	10 ng pCroatan-dualPromoter template
5 ul	10x KOD buffer (Millipore)
5 ul	DMSO
4 ul	$MgSO_4$
2 ul	KOD
X ul	H_2O
50 ul	Total volume

Put the PCR reaction in a thermocycler using the following program:

1. 95C for 5 mins
2. 95C for 30 s
3. 55C for 30 s
4. 72C for 30 s
5. Cycle to step 2 for 20 cycles
6. 72C for 30 s

Step 4. Purify the PCR reaction using the QIAquick PCR purification kit and digest using BbsI for 2 hours at 37C:

4 ug	PCR product
5 ul	10x NEBuffer 2.1 (NEB)
2 ul	BbsI (Thermo Fischer)
X ul	H_2O
50 ul	Total volume

Step 5. Gel purify the ~1.2kb PCR product containing the sgRNAs and the two U6 promoters using a QIAquick Gel Extraction Kit.

Step 6. Set up ligation and incubate at room temperature for 1 hour:

X ul	100 ng of digested pCRoatan
X ul	40 ng of digested PCR product
2 ul	10x T4 DNA ligase buffer (NEB)
1 ul	T4 DNA ligase (NEB M0202T)
X ul	H_2O
20 ul	Total volume

Step 7. Transform into competent cells. Recombination-deficient bacteria must be used to prevent plasmid recombination. We use Endura electrocompetent cells (Lucigen #60242-2) for this transformation. Plate the transformation on Amp-Zeo-LB low salt agar plates.
